# Acute Ischaemic Stroke in Patients Treated with Direct Oral Anticoagulants: Potential Causes, Clinical Characteristics, and Short-Term Outcomes

**DOI:** 10.1155/2024/2285722

**Published:** 2024-02-10

**Authors:** Katarzyna Sawczyńska, Ewa Włodarczyk, Aleksandra Pawlicka, Bartosz Kołodziejczyk, Paweł Wrona, Kamil Wężyk, Tomasz Homa, Paulina Sarba, Dominik Wróbel, Kaja Zdrojewska, Maria Sobolewska, Dawid Rolkiewicz, Agnieszka Slowik

**Affiliations:** ^1^Department of Neurology, University Hospital in Kraków, Kraków, Poland; ^2^Department of Neurology, Jagiellonian University Medical College, Kraków, Poland; ^3^Department of Anatomy, Jagiellonian University Medical College, Kraków, Poland; ^4^Department of Physiotherapy, Jagiellonian University Medical College, Kraków, Poland; ^5^University Hospital in Kraków, Kraków, Poland; ^6^Jagiellonian University Medical College, Faculty of Medicine, Kraków, Poland

## Abstract

**Introduction:**

Direct oral anticoagulants (DOAC) are the first-line treatment for primary and secondary acute ischaemic stroke (AIS) prevention in patients with nonvalvular atrial fibrillation (NVAF), but a significant percentage of patients develop AIS despite being treated with DOAC. As the number of DOAC-treated patients is growing, so is the number of patients with AIS on DOAC. The aim of the study was to assess the incidence of AIS with prestroke DOAC treatment among patients hospitalised in the University Hospital in Kraków, to analyse the clinical characteristics of AIS occurring in patients on DOAC, and to identify potential causes of treatment ineffectiveness in this group.

**Materials and Methods:**

In the study, we included all patients hospitalised in the Department of Neurology of the University Hospital in Kraków within one year (July 2022 to June 2023) with the diagnosis of AIS. The group was divided into two subgroups of patients with and without prestroke DOAC treatment. Based on medical files, we retrospectively analysed the profile of cardiovascular risk factors, stroke severity (assessed with National Institutes of Health Stroke Scale, NIHSS), use of causative stroke treatment and short-term outcomes (defined as NIHSS score, modified Rankin scale (mRS) score at discharge, in-hospital mortality, and secondary intracerebral haemorrhage among patients treated with mechanical thrombectomy, MT). Within the DOAC-treated subgroup, we looked for potential causes of AIS occurring despite DOAC treatment (valvular AF, poor adherence to treatment, underdosing, other prothrombotic conditions, aetiology of stroke other than thromboembolic, and drug-drug interactions).

**Results:**

In the study, we included 768 AIS patients. 109 (14.2%) had a history of prestroke DOAC treatment. A potential cause of DOAC treatment failure was identified in the majority of them (*n* = 63, 57.8%). Patients with prestroke DOAC treatment had worse functional condition before stroke and higher stroke severity on admission but similar short-term outcomes and similar short-term effects of treatment with MT. DOAC (+) and DOAC (-) patients had different profiles of cardiovascular risk factors and different factors associated with short-term outcome. *Conclusions and Clinical Implications*. A potential cause of AIS occurring in DOAC-treated patients can be identified in most cases and in many of them prevented.

## 1. Introduction

Direct oral anticoagulants (DOAC) include direct factor Xa inhibitors (rivaroxaban, apixaban, and edoxaban) and a direct thrombin inhibitor (dabigatran), all of them being used as a first-line treatment in ischaemic stroke prevention for patients with nonvalvular atrial fibrillation (NVAF) [1]. Other indications for DOAC treatment include venous thromboembolism [2].

Although significantly reduced, the risk of acute ischaemic stroke (AIS) in NVAF patients undergoing DOAC treatment is still present and is estimated to be 0.7%-2.3% per year, with higher risk values in secondary prevention [3]. Studies show that 20-36% of AIS in AF patients occur despite treatment with oral anticoagulants [4]. The potential causes of AIS occurring in DOAC-treated patients include noncompliance to treatment, inappropriate drug dosage, interactions with other medications, aetiology of stroke other than cardioembolic, residual risk, and treatment failure [5]. At the same time, patients developing AIS during DOAC therapy demand a different approach to treatment, as DOAC intake within preceding 48 hours is a contraindication for intravenous thrombolysis (IVT). However, in case of dabigatran, there is a possibility to reverse its anticoagulation effect using idarucizumab [6] and case reports describing the use of andexanet alfa before IVT in patients treated with direct factor Xa inhibitors are starting to appear [7].

Ischaemic strokes occurring in anticoagulated AF patients tend to be less severe [8] and have better outcomes [9, 10]. AIS in DOAC-treated patients seem to cause milder neurological deficit and be smaller in size compared to infarcts occurring during treatment with vitamin K antagonists (VKA) [11], and they also seem to result in better outcomes [3, 9]. The use of DOAC in secondary stroke prevention also seems to be more beneficial than VKA, because if a recurrent infarct occurs, it also tends to be smaller in size [12]. At the same time, the number of DOAC users increases and so does the absolute number of patients with AIS occurring during DOAC treatment [4]. Some retrospective studies even suggest that the real-life incidence of AIS in anticoagulated patients may be higher on DOAC than on VKA, thus contradicting the results of clinical trials [13].

## 2. Clinical Rationale for the Study

With growing numbers of patients receiving DOAC in primary and secondary stroke prevention, the number of cases of AIS occurring in DOAC-treated patients is also increasing [4, 8], making it important to continuously analyse their incidence, potential causes, clinical course, and outcomes.

The aim of our study was to assess the annual prevalence of acute ischaemic stroke (AIS) with prestroke DOAC treatment among patients hospitalised in the Department of Neurology of the University Hospital in Kraków (Poland), analyse the clinical characteristics of AIS occurring in patients on DOAC, and identify potential causes of treatment ineffectiveness in this group. We also aimed to compare profile of cardiovascular risk factors, stroke severity, and short-term outcomes and factors influencing in-hospital outcome in AIS patients with and without prestroke DOAC treatment.

## 3. Materials and Methods

The presented study is a retrospective medical documentation analysis, including all patients hospitalised in the Department of Neurology of the University Hospital in Kraków (Poland) within one year (from July 2022 to June 2023) with the diagnosis of acute ischaemic stroke (AIS). The group was divided into two subgroups of patients with and without prestroke DOAC treatment.

We performed a detailed analysis of the DOAC-treated subgroup. We noted the indications for DOAC treatment, the type of DOAC used, its dose (full or reduced, on-label vs. off-label reductions, and reasons for off-label reduction), time from last dose uptake to admission, and the patients' adherence to treatment. We looked for other potential causes of DOAC treatment failure: unrecognized valvular AF (presence of moderate to severe mitral stenosis in echocardiography or implanted prosthetic mechanical heart valve) [15], large vessel disease (carotid atherosclerosis found in carotid ultrasound as described below), lacunar stroke suggesting AIS in the course of cerebral small vessel disease (cSVD), other prothrombotic conditions (concomitant malignancy, acquired or hereditary thrombophilia), and interactions with other medications that have potential to lower DOAC plasma levels (especially strong CYP3A4 inducers: rifampicin, carbamazepine, phenobarbital, and phenytoin) [16]. We also analysed activated partial thromboplastin time (APTT) levels, if available within 12 hours from stroke onset.

From medical files of all the patients (with and without prestroke DOAC treatment), we gathered information on their age, biological sex, and profile of cardiovascular risk factors:

- Arterial hypertension (systolic blood pressure of ≥140 mmHg or diastolic blood pressure ≥ 90 mmHg at least in two different measurements after the first 3 days of hospitalisation and/or antihypertensive treatment prior to stroke onset and/or arterial hypertension diagnosed in previous medical history)
- Diabetes/prediabetes (diagnosed based on ESC criteria) [17]
- Dyslipidaemia (cholesterol level > 5.2 mmol/L or use of cholesterol-lowering treatment before stroke)
- Atrial fibrillation (found in previous medical history or diagnosed during hospitalisation based on electrocardiograms)
- Coronary artery disease (found in previous medical history or diagnosed during hospitalisation based on available electrocardiograms and/or laboratory tests)
- Congestive heart failure (found in previous medical history or diagnosed during hospitalisation based on clinical symptoms, laboratory tests, and/or echocardiography)
- History of ischaemic stroke/transient ischaemic attack (TIA)
- History of smoking during previous 10 years

In patients who underwent carotid ultrasound during hospitalisation, we noted the presence of carotid atherosclerosis (intima-media complex thickening and/or presence of atherosclerotic plaques, with stenoses > 50% considered hemodynamically significant). Prestroke functional neurological condition was assessed using modified Rankin scale (mRS). Neurological deficit on admission was assessed using National Institutes of Health Stroke Scale (NIHSS). Causative treatment with intravenous thrombolysis (IVT) and/or mechanical thrombectomy (MT) was noted. For patients treated with MT, we assessed the radiological effect of the procedure using modified treatment in cerebral ischaemia (mTICI) score, with full reperfusion defined as mTICI 2b–3. We analysed the incidence of secondary intracerebral haemorrhage (sICH) after MT. Short-term outcome was assessed using NIHSS and mRS scores at discharge and in-hospital mortality. Good functional outcome was defined as mRS 0–2.

We compared abovementioned data in groups of patients with and without prestroke DOAC treatment using PS Imago Pro 9.0 statistical programme. We presented categorical data as absolute counts and percentages and compared it between groups using chi-square test. We presented continuous data as median and interquartile range (IQR) due to its non-normal distribution (assessed using the Kolmogorov-Smirnov test) and compared it between groups using Mann–Whitney *U* test. The level of significance was defined as two-tailed *p* value of < 0.05. In both subgroups, factors influencing short-term outcomes (good clinical outcome at discharge and in-hospital mortality) were identified using univariate logistic regression model, with variables with *p* < 0.05 subsequently included in multivariate analysis.

The study was approved by the Jagiellonian University Bioethics Committee (decision number 1072.6120.118.2020 dated May 28, 2020) and conducted in accordance with the Declaration of Helsinki.

## 4. Results

The study included 768 patients with AIS hospitalised in our centre within 12 months. Among them, 109 (14.2%) had a history of prestroke DOAC treatment. Their characteristics are summarized in Table 1.

DOAC-treated patients were aged 38–98 with a median age of 77 years (IQR = 14). Sixty-six (60.6%) were female. Thirty-three (30.3%) were treated with dabigatran, 47 (43.1%) with rivaroxaban, and 29 (26.6%) with apixaban. No patients were treated with edoxaban, due to its unavailability in Poland at that time.

In 101 (92.7%) patients, the reason for DOAC treatment was atrial fibrillation (AF), in 4 (3.7%) history of venous thromboembolism, and 3 (2.8%) patients received low-dose rivaroxaban (5 mg per day) for cardiovascular prevention according to COMPASS trial results [18], and in 1 patient (0.9%), the reason for DOAC treatment was unknown.

### 4.1. Potential Causes of DOAC Treatment Failure

Time from last dose uptake to admission was <12 hours in 26 patients (23.9%; with one patient taking a DOAC dose after the onset of AIS symptoms), 12-24 hours in 10 patients (9.2%), 24-48 hours in 5 patients (4.6%), >48 hours in 15 patients (13.8%), and unknown in 53 patients (48.6%). That means that at least 33.0% took last DOAC dose < 24 hours before admission. In 8 patients (7.3%), DOAC was ceased by their doctor before a planned surgery.

Twenty-four patients (22.0%) took DOAC on a regular basis, and 29 (26.6%) confirmed that they took DOAC irregularly. In 56 (51.4%), the level of drug adherence was unknown.

Full DOAC dose was used by 49 (45.0%) patients and reduced dose by 53 (48.6%) patients, and in 7 patients (6.4%), the dose was unknown. Among 53 patients using reduced DOAC doses, 12 (22.6%) were using dabigatran, 26 (49.1%) rivaroxaban, and 15 (28.3%) apixaban. The reduction was on-label in 33 (62.3%) patients and off-label in 17 (32.1%) patients (2 (11.8%) using dabigatran, 6 (35.3%) using rivaroxaban, and 9 (52.9%) using apixaban), and 3 rivaroxaban patients (5.7%) received COMPASS trial doses.

The reasons for off-label underdosing were older age in 4 patients, anaemia and/or history of bleeding in 2 patients, malignancy in 2 patients, recent surgery in 2 patients (in one of them a minor one), renal insufficiency and history of bleeding in 1 patient, and intolerance of side effects in 1 patient, and in 5 patients, we could not identify any potential cause of underdosing based on available medical documentation.

Valvular AF was found in 3 patients (2.7%). Two patients (1.8%) were diagnosed with hereditary thrombophilia. Eight (7.3%) had a concomitant malignancy. Carotid ultrasound results were available in 98 patients, out of whom 87 (88.8%) had carotid atherosclerosis with hemodynamically significant stenoses present in 19 (19.4%) patients. Lacunar stroke, suggestive of cSVD mechanism of stroke, occurred in 4 patients (3.7%). Significant drug-drug interactions were found in 1 patient (0.9%), treated with rivaroxaban, who simultaneously took carbamazepine.

APTT within 12 hours from stroke onset was available in 73 patients, and it ranged from 20.9 to 69.9 seconds with a median of 31.6 seconds (IQR = 7.1). In 57 (78.1%) of them, it did not exceed the upper normal limit for our laboratory (36 seconds).

A potential cause of DOAC treatment failure (off-label underdosing, poor adherence to treatment, withdrawal of medication before a planned surgery, valvular AF, concomitant thrombophilia or malignancy, presumed cSVD mechanism of stroke, significant carotid artery stenoses, and/or interactions with other medications) was identified in 63 patients (57.8%).

### 4.2. Differences between Patients with and without Prestroke DOAC Treatment

Comparison of groups of patients with and without prestroke DOAC treatment is summarized in Table 2. When compared to patients without prestroke DOAC treatment, the DOAC-treated patients were significantly older (median of 77 (IQR = 14) vs. 71 (IQR = 17) years, *p* < 0.001) and more commonly were female (60.6% vs. 47.0%, *p* = 0.010). They had a different profile of cardiovascular risk factors, more commonly suffering from arterial hypertension (87.2% vs. 71.9%, *p* < 0.001), dyslipidaemia (44.0% vs. 20.2%, *p* < 0.001), atrial fibrillation (93.6% vs. 26.6%, *p* < 0.001), coronary artery disease (27.5% vs. 18.4%, *p* = 0.028), congestive heart failure (39.4% vs. 9.4%, *p* < 0.001), and more commonly had history of previous ischaemic stroke or TIA (33.0% vs. 12.4%, *p* < 0.001), while history of smoking was less prevalent in this group (12.8% vs. 27.2%, *p* = 0.002).

Prestroke functional neurological condition was worse in DOAC-treated patients (median of 1 (IQR = 2) vs. 0 (IQR = 0) points, *p* < 0.001). Stroke severity at onset was higher in the DOAC-treated group, with median NIHSS score on admission of 14 (IQR = 13) vs. 11 (IQR = 13) points (*p* = 0.042). For obvious reasons, DOAC (+) patients were more rarely treated with IVT (13.8% vs. 46.7%, *p* < 0.001), with similar percentage of patients treated with MT in both groups (52.3% vs. 44.3%, *p* = 0.146).

The effectiveness of reperfusion in MT-treated patients did not differ between groups (full reperfusion reached in 86.0 vs. 86.3%, *p* = 1.000), the incidence of sICH after MT was also similar (33.3% vs. 27.6%, *p* = 0.420), and there were no differences in mortality in MT-treated patients with and without prestroke DOAC treatment (5.3% vs. 7.9%, *p* = 0.492).

We observed a significantly higher discharge mRS score in DOAC-treated patients (median of 3 (IQR = 4) vs. 2 (IQR = 3) points, *p* = 0.029), although the percentage of patients with good functional outcome was similar in both groups (42.2% vs. 51.4%, *p* = 0.079). The in-hospital mortality rate did not differ significantly (11.1% vs. 8.7%, *p* = 0.468). In patients who survived the stroke, neurological deficit at discharge was also similar (median NIHSS 5 (IQR = 13) vs. 3 (IQR = 8) points, *p* = 0.195).

### 4.3. Factors Influencing Short-Term Outcome in Patients with and without Prestroke DOAC Treatment

In the DOAC (+) group, multivariate logistic regression analysis showed that the only independent factor associated with in-hospital mortality was NIHSS score on admission (OR = 1.103, 95% CI: 1.001-1216, *p* = 0.047), although the *R*^2^ value of the model being 0.082 indicates a small effect size. The independent factors associated with good functional outcome in this group were NIHSS score on admission (OR = 0.852, 95% CI: 0.775-0.936, *p* < 0.001), prestroke mRS (OR = 0.355, 95% CI: 0.182-0.693, *p* = 0.002), history of stroke/TIA (OR = 0.162, 95% CI: 0.042-0.628, *p* = 0.008), congestive heart failure (OR = 0.195, 95% CI: 0.057-0.664, *p* = 0.009), and reduced DOAC dose (OR = 0.287, 95% CI: 0.091-0.909, *p* = 0.034), with *R*^2^ value of this model being 0.595.

In the DOAC (-) group, independent factors associated with in-hospital mortality were age (OR = 1.060, 95% CI: 1.026-1.096, *p* < 0.001), the presence of significant carotid stenoses (OR = 3.686, 95% CI: 1.854-7.328, *p* < 0.001), and NIHSS score on admission (OR = 1.141, 95% CI: 1.082-1.204, *p* < 0.001), with *R*^2^ value for this model being 0.272. Independent factors associated with good functional outcome in the best fit model (*R*^2^ = 0.481) were age (OR = 0.956, 95% CI: 0.933-0.981, *p* < 0.001), NIHSS score on admission (OR = 0.816, 95% CI: 0.766-0.870, *p* < 0.001), prestroke mRS (OR = 0.451, 95% CI: 0.255-0.796, *p* = 0.006), and full reperfusion after MT (OR = 12.240, 95% CI: 4.117-36.388, *p* < 0.001).

## 5. Discussion

During the last decade, our knowledge on AIS in patients with preceding DOAC treatment has vastly expanded [19]. Although AIS can occur even despite sufficient anticoagulation, a number of causes for DOAC treatment failure have been identified and researched [4, 5]. Our study shows that a potential cause of AIS occurring in DOAC-treated patients can be identified in most cases and in many of them prevented. The percentage of DOAC-treated patients with identifiable AIS cause could be higher in real life, as the retrospective model of our study did not allow us to fully analyse all possible data and some information was missing in patients' medical files.

Poor adherence to treatment increases the risk of AIS in DOAC-treated patients [20]. A preclinical study's results suggest the possibility of a paradoxical prothrombotic state occurring after short-term withdrawal of dabigatran, which may potentially be an additional mechanism of AIS occurrence in noncompliant patients [21]. Research into the incidence of poor adherence among DOAC users gives mixed results. Some studies show overall great adherence to treatment [22]. On the contrary, a study by Tiili et al. showed noncompliance rate of DOAC-treated patients with AF who had already suffered AIS to be as high as 44% and associated with tertiary education, history of smoking, lack of heart failure, prior use of vitamin K antagonists, and history of more than one stroke [23]. In our group, poor adherence to treatment was documented in 26.6% of DOAC-treated patients and was most likely much higher, as in more than 50% the data on compliance was missing.

DOAC underdosing is a common situation in clinical practice, especially due to fear of bleeding complications [24]. According to some studies, off-label underdosing is found in about a third of DOAC-treated AF patients [25]. Higher risk of underdosing is associated with older age, congestive heart failure, arterial hypertension, history of minor bleeds, and low creatine clearance [26]. A study by Tütüncü et al. showed that among 239 DOAC-treated AIS patients, 21.8% was underdosed before stroke onset [27]. In a case-control study by Paciaroni et al. including 713 DOAC-treated AIS patients, 44.5% (317 patients) was treated with low-dose DOAC and 35% of those (111 patients) with off-label low doses. Underdosing was found to be the main factor increasing the risk of ischaemic events in DOAC-treated patients [28]. Studies on the impact of underdosing on stroke risk give mixed results. A recent meta-analysis showed that patients treated with inappropriately lower DOAC doses do not have lower bleeding risk or higher AIS risk but have higher all-cause mortality [26]. Another meta-analysis showed increased risk of ischaemic events (including AIS) and mortality in patients on nonrecommended low DOAC doses without impact on major bleeding risk [29]. Another systematic review with meta-analysis did not find significantly higher risk of AIS, thromboembolism, bleeding, nor death in patients on off-label reduced DOAC doses [30]. A study by Steinberg et al. showed that DOAC underdosing is associated with higher rates of cardiovascular hospitalisation [31]. In a study by Jung et al., stroke severity was higher in patients on underdosed DOAC compared to standard dose DOAC [32]. A study by Lee et al. showed that increase of stroke risk is bigger among underdosed patients than the decrease of bleeding risk [33]. In our group, use of reduced DOAC doses was an independent risk factor of bad functional outcome at discharge.

Aetiology of stroke other than cardioembolic is also common in DOAC-treated patients who develop AIS. In the RENo study, stroke aetiology different than cardioembolic was found in 32.7% of AIS patients with prestroke DOAC treatment [28]. In a study by Polymeris et al., it was 24.2%, with the most common aetiology being, similar to our study, large artery atherosclerosis [3]. The impact of comorbidities may also be significant. A study by Lin et al. showed that patients with recurrent AIS despite DOAC therapy were more likely to suffer from a malignancy [34]. A study by Suda et al. showed that high levels of B-type natriuretic peptide, suggestive of congestive heart failure, were independently associated with AIS or TIA despite the use of oral anticoagulants (DOAC or VKA) [35].

Medications inducing CYP3A4 or P-glycoprotein have the potential to reduce plasma levels of DOAC [16]. Strong CYP3A4 inducers include rifampicin, phenytoin, phenobarbital, and carbamazepine [36]. In a study by Lin et al., patients with recurrent stroke despite DOAC treatment were more often treated with CYP3A4-inducing antiepileptic medications [34]. Still, there are not many literature reports of adverse events occurring due to drug-drug interactions (DDI) of DOAC, and what is more, DOAC have fewer interactions than VKA [36]. In our group, significant DDI were also quite rare, found in 1 patient (0.9%).

Different profile of cardiovascular risk factors in DOAC-treated patients and in the DOAC (-) group is most likely related to comorbidity of atrial fibrillation, being the most common cause of DOAC use in our group and present in the majority of patients (96.3%). More prevalent history of stroke or TIA and smaller percentage of smokers among DOAC-treated AIS patients compared to patients without prestroke history of DOAC were already observed in previous studies [8].

In our group, there was no difference in short-term MT outcomes (sICH, in-hospital mortality, and good functional outcome at discharge) between patients with and without prestroke DOAC treatment. Previous studies on the effect of MT in anticoagulated patients give mixed results. In a study by Çabalar et al., patients using anticoagulants (DOAC or VKA) prior to stroke onset had higher rates of successful recanalization than nonanticoagulated patients [37]. In another study by Küpper et al., there was no difference in successful recanalization rate in MT-treated patients with and without prior anticoagulation, and although 90-day functional outcome was worse in anticoagulated patients, logistic regression analysis adjusted for other clinical data showed that the impact of prior anticoagulation on 90-day functional outcome was not statistically significant [38]. Another study by Nowak et al. showed no differences in long-term outcomes of MT in patients with and without prestroke anticoagulation [39]. A systematic review with meta-analysis by Liu et al. showed no differences between MT-treated patients with and without prestroke anticoagulation in the occurrence of sICH, full recanalization, and in-hospital mortality, but patients with prior anticoagulant use had worse functional outcome [40].

In our study, the severity of AIS at onset was higher in DOAC (+) than DOAC (-) patients, which would be consistent with previous studies on the impact of preceding anticoagulation on stroke severity [8], but our results may have been affected by the fact that our control group consisted of all other patients with AIS hospitalised in our centre, including VKA-treated patients. The same thing may have flawed our comparison of short-term outcomes between the subgroups.

Still, the most important limitation of our study was its retrospective design, not allowing us to analyse many factors due to lack of data, such as the duration of DOAC treatment (AIS in patients receiving DOAC is reported to occur mostly during the first few months of treatment [11]), reasons for poor adherence to treatment, or rivaroxaban intake with or without food (the drug should be taken together with a meal to increase its plasma level [16]). The most interesting groups of patients are those who develop AIS despite sufficient anticoagulation, having no other potential stroke cause than cardioembolic. Analysing this subgroup was unfortunately beyond the scope of this study. Because in some patients, some data was missing (i.e., due to their severe neurological condition or aphasia) that it was impossible for us to identify all of such cases. Another important limitation is the relatively small number of DOAC (+) cases, causing troubles with identifying factors associated with mortality and functional outcome using multivariate logistic regression. We also analysed only short-term outcomes of DOAC-treated AIS patients. More prospective studies are needed on this matter, and we can expect more data to come from ongoing trials, including the ARAMIS registry [41].

## 6. Clinical Implications/Future Directions

De Magistris and Paciaroni gave valuable suggestions on how to manage patients with recurrent stroke despite DOAC treatment, which includes excluding poor adherence, analysing the DOAC dose, looking for drug-drug interactions, searching for an alternative stroke aetiology, considering further treatment (the same DOAC, other DOAC, DOAC+antiplatelet, and left atrial appendage occlusion), and continuing research on this topic [5]. Our study shows that in the majority of cases, the reason for DOAC treatment failure can be identified and often prevented, so we encourage clinicians to actively screen patients treated with DOAC in both primary and secondary stroke prevention for factors affecting the effectiveness of this treatment, especially the reversible ones such as noncompliance, inappropriate dosing, or DDI.

## Figures and Tables

**Table 1:** Characteristics of DOAC-treated subgroup.

Personal information	
Age (median (IQR))	77 (IQR 38–98)
Female sex (*n* (%))	66 (60.6%)
DOAC	
Dabigatran (*n* (%))	33 (30.3%)
Rivaroxaban (*n* (%))	47 (43.1%)
Apixaban (*n* (%))	29 (26.6%)
Reason for DOAC treatment	
AF (*n* (%))	101 (92.7%)
Thromboembolism (*n* (%))	4 (3.7%)
COMPASS trial dose (*n* (%))	3 (2.7%)
Unknown (*n* (%))	1 (0.9%)
The last dose of DOAC before admission (*n* (%))	
<12 hours	26 (23.9%)
12-24 hours	10 (9.2%)
24-48 hours	5 (4.6%)
>48 hours	15 (13.8%)
Unknown	53 (48.6%)
Compliance (*n* (%))	
Full compliance	24 (22.0%)
Noncompliance	29 (26.6%)
Unknown	56 (51.4%)
DOAC dose	
Full (*n* (%))	49 (45%)
Reduced (*n* (%))	53 (48.6%)
Dabigatran	12 (22.6%)
Rivaroxaban	26 (49.1%)
Apixaban	15 (28.3%)
On-label underdosing	33 (62.3%)
Off-label underdosing	17 (32.1%)
	2 (11.8%) = dabigatran
	6 (35.3%) = rivaroxaban
	9 (52.9%) = apixaban
COMPASS trial rivaroxaban dose	3 (5.7%)
Unknown (*n* (%))	7 (6.4%)
A potential cause of DOAC treatment failure (*n* (%))	63 (57.8%)
Valvular AF (*n* (%))	3 (2.7%)
Hereditary thrombophilia (*n* (%))	2 (1.8%)
Concomitant malignancy (*n* (%))	8 (7.3%)
Carotid atherosclerosis with hemodynamically significant stenoses (*n* (%))	19 (19.4%)
Lacunar stroke (*n* (%))	4 (3.7%)
Significant drug-drug interactions (*n* (%))	1 (0.9%)
APTT	
Available APTT < 12 h from stroke onset (*n* (%))	73 (66.9%)
APTT (median (IQR))	31.6 (IQR 20.9–69.9) seconds

DOAC = direct oral anticoagulants; AF = atrial fibrillation; APTT = activated partial thromboplastin time.

**Table 2:** Comparison of patients with and without prestroke DOAC treatment.

	DOAC (+)	DOAC (-)	*p*
Risk factors			
Age, years (median (IQR))	77 (IQR = 14)	71 (IQR = 17)	<0.001
Female sex (*n* (%))	66 (60.6%)	310 (47.0%)	0.010
Arterial hypertension (*n* (%))	95 (87.2%)	474 (71.9%)	<0.001
Diabetes/prediabetes (*n* (%))	81 (74.3%)	509 (77.2%)	0.540
Dyslipidaemia (*n* (%))	48 (44.0%)	133 (20.2%)	<0.001
Significant carotid stenosis (*n* (%))^1^	19 (19.4%)	144 (24.5%)	0.306
Atrial fibrillation (*n* (%))	102 (93.6%)	175 (26.6%)	<0.001
Coronary artery disease (*n* (%))	30 (27.5%)	121 (18.4%)	0.028
Congestive heart failure (*n* (%))	43 (39.4%)	62 (9.4%)	<0.001
History of stroke/TIA (*n* (%))	36 (33.0%)	82 (12.4%)	<0.001
History of smoking (*n* (%))	14 (12.8%)	179 (27.2%)	0.002
Disease course			
Prestroke mRS (median (IQR))^2^	1 (IQR = 2)	0 (IQR = 0)	<0.001
NIHSS on admission (median (IQR))	14 (IQR = 13)	11 (IQR = 13)	0.042
IVT (*n* (%))	15 (13.8%)	308 (46.7%)	<0.001
MT (*n* (%))	57 (52.3%)	292 (44.3%)	0.146
MT outcomes			
Full reperfusion (TICI 2b-3) (*n* (%))	49 (86.0%)	252 (86.3%)	1.000
sICH after MT (*n* (%))	19 (33.3%)	72 (27.6%)	0.420
Mortality	3 (5.3%)	23 (7.9%)	0.492
Stroke outcomes			
Discharge mRS (median (IQR))^3^	3 (IQR = 4)	2 (IQR = 3)	0.029
Good functional outcome (*n* (%))^3^	46 (42.2%)	335 (51.4%)	0.079
Discharge NIHSS (median (IQR))^4^	5 (IQR = 13)	3 (IQR = 8)	0.195
Mortality (*n* (%))	12 (11.1%)	57 (8.7%)	0.468

^1^Data available in 98 DOAC (+) and 587 DOAC (-) patients. ^2^Data available in 109 DOAC (+) and 605 DOAC (-) patients. ^3^Data available in 109 DOAC (+) and 652 DOAC (-) patients. ^4^Data available in 97 DOAC (+) and 596 DOAC (-) patients.

## Data Availability

The data that support the findings of this study are available from the corresponding author, Katarzyna Sawczyńska, upon reasonable request.
